# Cheilitis caused by contact allergy to toothpaste containing stannous (tin) – two cases

**DOI:** 10.1111/cod.13532

**Published:** 2020-04-13

**Authors:** Cynthia C.A. van Amerongen, Anton de Groot, Rob J. Volkering, Marie L.A. Schuttelaar

**Affiliations:** ^1^ Department of Dermatology, University of Groningen University Medical Center Groningen Groningen The Netherlands; ^2^ Schipslootweg Wapserveen The Netherlands

**Keywords:** allergic contact dermatitis, case report, cheilitis, contact allergy, stannous chloride, stannous fluoride, stannous oxalate, tin, toothpaste

Allergic contact cheilitis can be caused by contact allergy to different toothpaste ingredients. We report two patients with contact allergy to tin present as an ingredient in toothpaste.

## CASE REPORT

### Case 1

**FIGURE 1:**
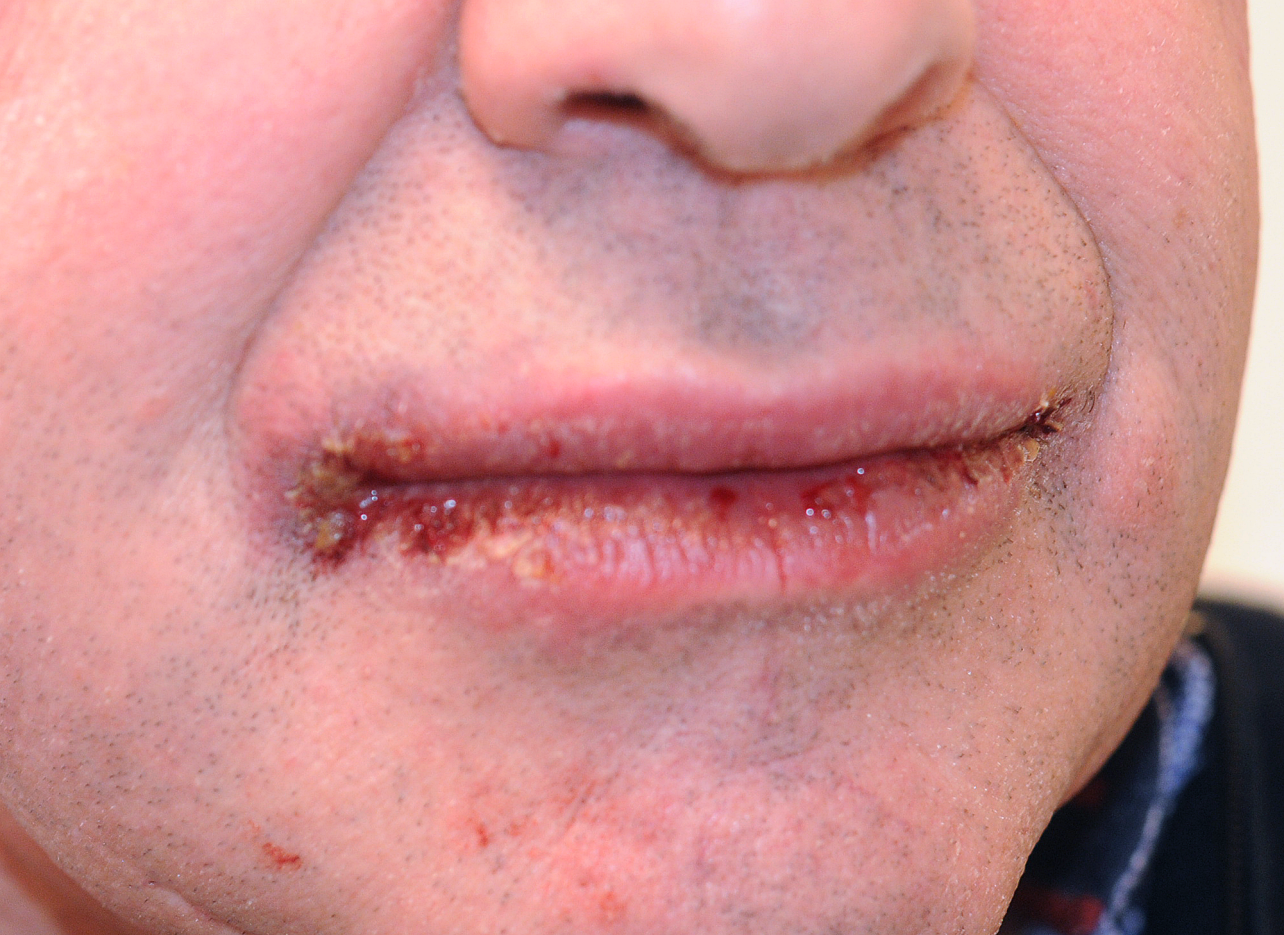
Erythematous plaques with crusts located on the lips in patient 1

A 69‐year‐old atopic man (retired painter) was referred for evaluation of cheilitis. He reported recurrent swelling with small blisters and red spots intra‐orally and on his tongue and, in addition, crusts on his lips for 6 months (Figure 1). He was using Sensodyne Rapid Relief toothpaste (GlaxoSmithKline, Brentford, UK).

### Case 2

A 62‐year‐old non‐atopic housewife was referred for evaluation of erythematous gingiva. At consultation she recalled episodes of red and flaking skin changes on and below her lip. She switched from Sensodyne Rapid Relief toothpaste to Urtekram *Aloe Vera* toothpaste (Urtekram, Mariager, Denmark), after which improvement of her complaint occurred. Unfortunately, after a few months, this toothpaste also started to cause peri‐oral and gingival symptoms.

#### Patch testing and results

Both patients were patch tested with our extended European baseline series (TRUE Test panels 1 and 2, supplemented with additional investigator‐loaded allergens), as well as a dental and metal series. Patient 2 was also tested with a cosmetics and fragrance series (allergens from SmartPractice Europe, Barsbüttel, Germany and Chemotechnique Diagnostics, Vellinge, Sweden). Van der Bend Chambers (Van der Bend, Brielle, The Netherlands) were applied on the back for 48 hours under occlusion and were fixed with Fixomull Stretch (BSN Medical, Hamburg, Germany). Patch test readings were performed at day (D) 3 and D7.

The results of patch testing are shown in Table 1. Patient 1 showed a positive reaction (+) to tin 50% pet. at D3 and D7. Tin 50% pet. was retested and gave a positive reaction (+) at D3 reaction and a strong positive reaction (++) at D7. Patient 2 showed a positive reaction (+) at D3 and a strong positive reaction (++) at D7 to tin 50% pet. Also, a positive reaction (+) to hydroperoxides of limonene 0.3% pet. at D3 was found, which became negative at D7. Examination of the Sensodyne Rapid Relief toothpaste's ingredients revealed the presence of stannous (tin) fluoride.

**TABLE 1:** Patch test results of two cases of allergic contact cheilitis

Tested series	Concentration, vehicle	Case 1				Case 2			
		Day 3		Day 7		Day 3		Day 7	
Metal series									
1st test: Tin^a^ ^− CAS 7440‐31‐5^	50% pet.	+		+		+		++	
2nd test: Tin^a^ ^− CAS 7440‐31‐5^	50% pet.	+		++		NT		NT	
Fragrance series									
Hydroperoxides of limonene	0.3% pet.	NT		NT		+		‐	
Toothpaste product									
Sensodyne Rapid Relief toothpaste	"as is"	+++		+++		+++		+	
	50% aqua	++		++		+		+	
	30% aqua	++		++		NT		NT	
	10% aqua	++		++		?+		+	
	5% aqua	NT		NT		−		−	
	3% aqua	+		+		−		−	
Urtekram *Aloe vera* toothpaste	"as is"	NT		NT		+		+	
	50% aqua	NT		NT		+		+	
	10% aqua	NT		NT		+		+	
	5% aqua	NT		NT		+		+	
	3% aqua	NT		NT		?+		+	
Additional substances									
Tin^b^ ^‐ CAS 7440‐31‐5^	30% pet.	−		−		++		+	
	10% pet.	−		−		+		+	
	3% pet.	−		−		−		−	
	1% pet.	−		−		−		−	
Stannous oxalate^a^ ^− CAS 814–94‐8^	1% pet.	++		++		++		++	
Stannous chloride^a^ ^− CAS 7772‐99‐8^	1% pet.	+++		++		+++		+++	
		**Pet.**	**Aqua**	**Pet.**	**Aqua**	**Pet.**	**Aqua**	**Pet.**	**Aqua**
Stannous fluoride^b^ ^− CAS 7783‐47‐3^	0.5% pet. and aqua	++	+	++	+	+++	?+	+++	+
	0.15% pet. and aqua	+	−	+	+	++	+	++	+
	0.05% pet. and aqua	+	−	+	−	++	−	++	−
	0.015% pet. and aqua	−	−	−	−	?+	−	+	−
Sodium fluoride^c^ ^– CAS 7681‐49‐4^	0.5/0.15/0.05/0.015% pet. and aqua	−	−	−	−	−	−	−	−

Abbreviations: NT, not tested.

a Chemotechnique Diagnostics.

b Raw material from Sigma‐Aldrich chemistry dilution series were prepared at the University Medical Centre Groningen (UMCG) pharmacy.

c Raw material from Duchefa Farma, dilution series were prepared in the UMCG pharmacy.

Both patients were then patch tested with the Sensodyne Rapid Relief toothpaste “as is” and in a dilution series (50%, 30%, 10%, 5%, and 3% aq.), which yielded extreme positive reactions (+++) at D3 and D7 in patient 1 and at D3 in patient 2. Patient 1 showed positive reactions throughout the entire dilution series and patient 2 down to the 10% concentration. Patient 2 was also tested with Urtekram *Aloe vera* toothpaste and showed positive reactions (+) to pure toothpaste and all dilutions. Additional testing was also performed with a dilution series of tin (30%, 10%, 3%, and 1% pet.) and stannous fluoride (0.5%, 0.15%, 0.05%, and 0.015% in pet. and aq.) (raw material from Sigma‐Aldrich Chemistry, Darmstadt, Germany; dilution prepared at the University Medical Center, Groningen [UMCG]), stannous chloride 1.0% pet. and stannous oxalate 1% pet. (Chemotechnique Diagnostics); see Table 1. To exclude sodium fluoride as being the culprit allergen, sodium fluoride was tested (raw material from Duchefa Farma [Haarlem, The Netherlands], dilution 0.5%, 0.15%, 0.05%, and 0.015% in pet. and aq. prepared at the UMCG), which gave no positive reactions in either patient.

Three controls were tested with Sensodyne Rapid Relief toothpaste who all showed negative reactions. In 38 controls tested with stannous chloride 1.0% pet., two strong positive (++), five weak positive (+), three doubtful and seven irritant reactions were seen. Of 38 controls tested with stannous oxalate 1% pet., one positive reaction was seen; this subject also reacted positive (+) to stannous chloride. These reactions were clinically relevant in this subject, because she had periorbital eczema and tin was found in her eye shadow. After discontinuation of the use of the eye shadow her periorbital eczema resolved completely. Of these controls, 28 subjects were also tested with tin 50% pet. and showed no positive reactions.

## DISCUSSION

We have described two patients with allergic contact cheilitis caused by tin present in Sensodyne Rapid Relief toothpaste (www.sensodyne.nl), which contains 0.454% w/w tin in the form of stannous (tin) fluoride. Stannous fluoride (CAS no. 7783‐47‐3) is a chemical compound that can be found as an ingredient in toothpaste.^1^ Fluorides are considered to be the most active ingredient in toothpaste with beneficial effects on caries, dental plaque, gingivitis, and halitosis.^1^, ^2^ It can also be beneficial for dentin hypersensitivity and is used for tubule occlusion causing nerve desensitization.^2^, ^3^

Patient 1 had used this toothpaste for over 20 years without complaints. After the diagnosis of contact allergy to tin was made, he switched to a toothpaste without tin and 2 weeks afterwards all skin complaints had resolved. Patient 2 had already switched from Sensodyne Rapid Relief toothpaste to Urtekram *Aloe vera* toothpaste with initial improvement of her gingival and skin complaints; however, these later recurred. This may well have been caused by her allergy to hydroperoxides of limonene; limonene was found to be an ingredient of the Urtekram toothpaste (www.urtekram.nl). After discontinuation of this toothpaste, the patient's peri‐oral and gingival symptoms resolved completely.

Contact allergy to toothpaste and its ingredients has been critically reviewed by de Groot.^2^ Symptoms usually manifest as cheilitis and dermatitis around the mouth can be present as well. Intra‐oral symptoms are less common. Even though cheilitis is the main symptom of contact allergy to toothpaste ingredients, toothpastes may be under‐recognized as a potential cause of cheilitis,^4^ possibly because contact allergy to toothpastes is infrequent.^2^ In the literature, flavouring agents are the most frequently reported cause of toothpaste allergy.^2^ Of the 34 critically reviewed reports by de Groot^2,^ only one case report described contact allergy caused by stannous fluoride.^5^ However, the toothpaste itself was not tested and no controls were included.^5^ Recently, another case with allergic contact dermatitis caused by stannous fluoride in toothpaste has been reported.^6^

In the literature, little is known about testing with tin salts. Olivarius et al reported 2206 patients patch tested with stannous chloride 1% pet. among whom 0.2%, 0.7%, and 0.6% patients showed positive, doubtful, and irritant reactions, respectively.^7^ We showed a high number of positive (7/38, 18%), doubtful (3/38, 8%), and irritant (7/38, 19%) reactions to stannous chloride 1%. In all but one control, there was no clinical relevance. It seems, therefore, that the 1% patch test concentration of stannous chloride is too high, given the frequently observed false‐positive and irritant reactions. Nevertheless, we feel confident that in both our patients with cheilitis, contact allergy to tin is certain, based on all the other positive reactions to tin and tin salts. We suggest testing with the commercially available stannous oxalate 1% pet. when suspecting tin contact allergy. In both our patients strong (++) positive reactions were seen to stannous oxalate 1% pet. and in only one control a ‐ relevant ‐ positive reaction was found.

## CONFLICTS OF INTEREST

The authors declare no conflicts of interest.

## AUTHOR CONTRIBUTIONS

**Cynthia van Amerongen:** Conceptualization; investigation; methodology; writing‐original draft; writing‐review and editing. **Anton de Groot:** Methodology; writing‐review and editing. **Rob Volkering:** Investigation; writing‐review and editing. **Marie Schuttelaar:** Conceptualization; investigation; methodology; supervision; writing‐review and editing.
